# Working Memory and Decision Processes in Visual Area V4

**DOI:** 10.3389/fnins.2013.00018

**Published:** 2013-02-26

**Authors:** Benjamin Y. Hayden, Jack L. Gallant

**Affiliations:** ^1^Department of Molecular and Cell Biology, University of California BerkeleyBerkeley, CA, USA; ^2^Department of Psychology, University of California BerkeleyBerkeley, CA, USA; ^3^Helen Wills Neuroscience Institute, University of California BerkeleyBerkeley, CA, USA

**Keywords:** matched filter, attention, feature-based attention, rapid serial visual presentation, delayed match-to-sample

## Abstract

Recognizing and responding to a remembered stimulus requires the coordination of perception, working memory, and decision-making. To investigate the role of visual cortex in these processes, we recorded responses of single V4 neurons during performance of a delayed match-to-sample task that incorporates rapid serial visual presentation of natural images. We found that neuronal activity during the delay period after the cue but before the images depends on the identity of the remembered image and that this change persists while distractors appear. This persistent response modulation has been identified as a diagnostic criterion for putative working memory signals; our data thus suggest that working memory may involve reactivation of sensory neurons. When the remembered image reappears in the neuron’s receptive field, visually evoked responses are enhanced; this match enhancement is a diagnostic criterion for decision. One model that predicts these data is the matched filter hypothesis, which holds that during search V4 neurons change their tuning so as to match the remembered cue, and thus become detectors for that image. More generally, these results suggest that V4 neurons participate in the perceptual, working memory, and decision processes that are needed to perform memory-guided decision-making.

## Introduction

Recognizing items held in short-term memory is a sophisticated cognitive process that requires the coordination of perception, working memory, and decision-making (Desimone, 1996; Romo and Salinas, 2003). It is typically believed that constituent elements of cognition are mediated by distinct specialized neural substrates (e.g., Meyer et al., 1988; Sigman and Dehaene, 2005). In the case of memory-guided decisions, it is argued that a working memory trace of task-relevant stimuli is maintained in the response patterns of neurons in specific regions of the lateral prefrontal cortex, and that this trace is compared with a representation of the visual stimulus in other prefrontal structures (Fuster and Alexander, 1971; Funahashi et al., 1989; Goldman-Rakic, 1995; Miller et al., 1996; Rainer et al., 1998). However, some evidence suggests that working memory may involve reactivation of sensory neurons (Ferrera et al., 1994; Zhou and Fuster, 1996; Super et al., 2001; Lee et al., 2005; Pasternak and Greenlee, 2005; Harrison and Tong, 2009; Lui and Pasternak, 2011; Riggall and Postle, 2012). This evidence suggests that changes in the tonic response properties of sensory neurons may instantiate a memory trace. Certain models of choice suggest that neurons that serve as the site of storage may also be involved in the decision (Machens et al., 2005). Thus, the same sensory neurons that initially respond to a stimulus may also maintain it in working memory and participate in the decision about whether the stimulus matches one held in memory.

To investigate the neural mechanisms of memory-guided decision-making we recorded neuronal responses in visual area V4 during a demanding match-to-sample task that involved selective spatial and feature-based attention and short-term maintenance of a cued image (the same dataset was analyzed, testing different hypotheses, in Hayden and Gallant, 2005, 2009; David et al., 2008). Subjects were trained to respond when a stimulus matching a cue presented at the beginning of the trial reappeared in a rapid serial visual presentation (4 Hz) of images presented at a location several degrees from the center of fixation. Our previous results suggest that task demands can shift the tuning of V4 neurons to match the searched-for cue (Mazer and Gallant, 2003; David et al., 2008). These results therefore suggest that V4 may instantiate a *matched filter*, which creates a representation of the remembered stimulus in the form of changes to the tuning properties of the neuron (Mazer and Gallant, 2003; David et al., 2008; see also Motter, 1994; Mirabella et al., 2007). A matched filter mechanism is efficient because it uses the same neurons to process sensory information, store memories, and guide decisions (Machens et al., 2005; Miller and Wang, 2006; David et al., 2008; Jun et al., 2010). The idea that individual V4 neurons instantiate a matched filter is also consistent with many neurophysiological, psychophysical, and theoretical studies (Olshausen et al., 1993; Lee et al., 1999; Rao and Ballard, 1999; Cutzu and Tsotsos, 2003; Carrasco et al., 2004; Lu and Dosher, 2004; Machens et al., 2005; Compte and Wang, 2006; Mirabella et al., 2007; David et al., 2008).

We observed three patterns consistent with the matched filter model. *First*, response rates observed during the delay period between the cue and the subsequent probes depend on the identity of the remembered stimulus. Like putative working memory effects observed in the prefrontal cortex, these changes in response rate persist across both the delay and the presentation of non-matching (and thus behaviorally irrelevant) probe images (Funahashi et al., 1989; di Pellegrino and Wise, 1993; Miller and Desimone, 1994; Goldman-Rakic, 1995; Miller et al., 1996; Romo et al., 2002). *Second*, the visually evoked responses to remembered images when they reappear in the stream of distractors, in the context of a match, are stronger than responses to other images. This response is a form of match enhancement (cf. Ogawa and Komatsu, 2004; Ogawa and Komatsu, 2006; Mirabella et al., 2007). *Third*, responses to remembered images that appear at the unattended location are different from responses to other distractors, suggesting that tuning shifts apply to neurons across the visual field. This effect confirms that response modulations are not simply an efferent motor signal. Collectively, our data are consistent with the hypothesis that attention can alter response properties of sensory neurons beyond simple gain changes in order to facilitate tasks like memory-guided decision-making (David et al., 2008).

## Materials and Methods

### Subjects and physiological procedures

The data reported in this paper were originally collected for another purpose and were published as part of other studies (Hayden and Gallant, 2005, 2009; David et al., 2008). However, the analyses presented here are all new. All animal procedures were approved by oversight committees at the University of California, Berkeley and satisfied or exceeded all NIH and USDA regulations. Methods have been reported in detail elsewhere (Mazer and Gallant, 2003). In brief, extracellular single-neuron recordings were performed with epoxy-coated tungsten electrodes (FHC, Bowdoinham, ME, USA) from two macaques (*Macaca mulatta*). Neural signals were amplified, band-pass filtered, and isolated with a spike sorter (Plexon Instruments, Dallas, TX, USA). Area V4 was located anatomically by exterior cranial landmarks and/or by direct visualization of the lunate sulcus, and confirmed by comparing receptive field properties to those reported previously.

Eye position was monitored with an infrared eye tracker (500 Hz: Eyelink II, SR Research, Toronto, CA, USA). Small changes in eye position in different trial conditions may lead to artifactual changes in response rate. We therefore excluded from further analyses all trials during which eye position deviated by more than 0.5° from the fixation spot (between 15 and 30% of trials, depending on the session). To verify that the observed effects did not result from subtle difference in eye position within this fixation window in the different task conditions, we compared eye position in the different conditions. We found that eye position does not depend on either the match image used or the cued spatial direction for any neuron in our data set (a randomized *t*-test was performed for all individual sessions, *p* > 0.05 in all cases).

We first estimated the boundaries of each classical receptive field (CRF) by hand. We then confirmed these manual estimates by an objective spatial mapping procedure that used reverse correlation of neural responses against a dynamic sequence of squares flashed in and around the CRF. We only recorded from neurons in which the manual and objective receptive field estimates were in good agreement. Furthermore, we avoided recording from any neurons whose CRF overlapped or approached within 4° of the fixation point (and thus the fovea). CRF diameters ranged from 3° to 8° (median 5°) and eccentricities ranged from 7° to 20° (median 12°).

### Behavioral task

**Figure 1:**
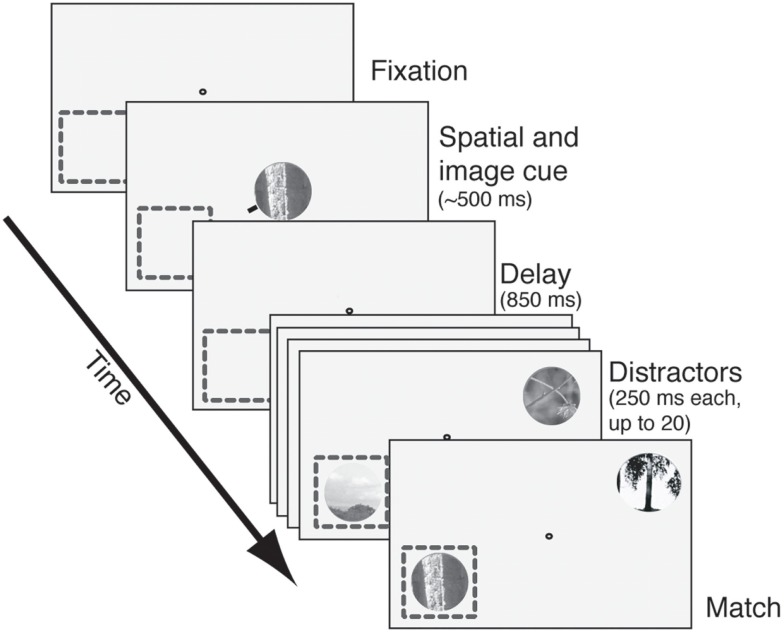
**The delayed match-to-sample task**. After fixation was acquired, an image cue was presented centrally. A small red line (spatial cue) designated the relevant search stream. After a delay period (850 ms in most cells, 350 ms in the rest), two streams of images appeared. One stream appeared in the receptive field (dashed square) and the other appeared in the opposite quadrant. Images appeared at approximately 4 Hz with no blank interval between successive images. Reward was given for bar release within 1 s after the match appeared in the cued stream. Failures to release, early releases, and fixation breaks at any time were considered errors. The match and all distractors were circular patches selected from photos and fit to the size of the neuron’s receptive field.

The task we used is illustrated in Figure 1; an illustrative video is found in the Supplement. Trials began when subjects grabbed a capacitive touch bar. A fixation spot then appeared. After fixation was acquired an image cue (natural image patch) and spatial cue (small red line pointing toward one location) appeared for 150–600 ms. (For approximately half the cells the spatial cue only appeared on the first trial in the block.) Care was taken to ensure that the cues never encroached upon the CRF. Following an 850 ms blank period (350 ms in the first set of 30 neurons recorded) two stimulus streams appeared: one in the CRF and one 180° away, in the opposite hemifield. Image patches appeared at a constant rate (3.5–4.5 Hz, varying across cells) and there was no blank period between successive stimuli. To receive reward subjects had to maintain continuous fixation and release the response bar within 1 s of the appearance of the match in the cued stream.

The stimuli were circular patches digitally cut out of black and white digital photographs of natural scenes (Corel Corp.) and blended into a gray background. The initial image library contained more than 100,000 images. A random pair of these was chosen as the set of distractors for each recording session. During the course of a recording session each neuron was probed with about 2,000–4,000 distinct images, and the set of images used to probe each neuron were largely independent. We made no effort to repeat presentation of any specific image across neurons, although they were sampled with replacement. Images were chosen by an automated algorithm that selected them at random, but that favored images with broad spatial frequency spectra. All images, both distractors and cues, were chosen without regard to neuronal tuning. Each image was approximately the same size as the CRF. Images were not normalized for contrast or luminance. At the beginning of each day two target images were chosen arbitrarily from the set of all images. To avoid any long-term bias, no image served as a target on more than 1 day. We later compared the average contrast, luminance, and power spectrum of these match images to those of the distractors. They did not differ statistically.

We presented two streams of images, one inside the receptive field and one opposite. On half the trials, the receptive field location was cued (we call this the attend-in condition). On the other half of the trials, the opposite location was cued (we call this the attend-out condition). On each trial, one of two images was cued by showing it centrally at the beginning of the trial. On half the trials one image was cued; on the other half, the other image was cued. Image and spatial attention were independent and we fully crossed these two attention states, creating four (2 × 2) crossed condition. This design allowed us to measure the main effect of spatial and feature (i.e., image) attention, as well as their interactions.

These conditions were run in blocks of 10 trials. Each block was associated with a single combination of spatial and image conditions. Thus, there were four types of 10-trial blocks, which were run in a specific sequence. The spatial cue alternated each block; the image cue alternated every two blocks. The entire sequence thus repeated every four blocks, or 40 trials. We collected a minimum of 200 trials per neuron, so we collected a minimum of five repeats of the 40-trial block sequences. Thus it is unlikely that any effects we observed reflect instabilities in neuronal isolation over time. On any trial as many as 20 distractor images could appear before the match. To ensure that the subject did not adopt a strategy of responding to either target (which would have required remembering both targets), the uncued image appeared in the cued stream with the same frequency as the match. In this case it was called the feature catch image. To ensure that the subject did not adopt a strategy of responding to the match when it appeared in either stream, the match was shown in the uncued stream (spatial catch image). The feature catch image and spatial catch were shown with approximately the same probability as the match. Specifically, on each trial, a random number generator chose a position in the sequence from 1 to 20 for each of the match, the spatial, and the feature catch; the cue then appeared at this position in the sequence at the appropriate spatial position. Responses to the catches caused the trial to end immediately and were not rewarded. The uncued image was never presented at the uncued location.

### Data analysis

Only data from correct trials were analyzed. To calculate the attention-dependent changes in undriven firing rate during the delay (i.e., the delay modulation), activity was averaged between the time of the disappearance of the cue and the appearance of the first distractor. Responses were averaged over the two spatial conditions. The analysis for image cue-related delay modulation was repeated for each of the two spatial conditions separately; significant modulations were found for both spatial conditions. We observed no interaction between the spatial and match image memory effects; these results are not reported here.

To calculate match enhancement, activity was compared in a window from 50 to 300 ms after the appearance of the image. Response rates in different conditions were compared with a two-tailed randomized *t*-test. For any two conditions, the distribution of responses expected by chance was determined by randomly assigning responses to the two conditions 1,000 times. An observed modulation was accepted as significant if it was greater than 97.5% or smaller than 2.5% of the randomized distribution (i.e., two-tailed *t*-test with *p* < 0.05).

In our timing analysis of match enhancement, comparison of the match and catch responses was restricted to a 50 ms window centered on the time of the peak of the transient response. Note that we chose to focus on the enhancement of matches relative to catches, rather than to distractors, because catches and matches are fully controlled for visual properties, thus isolating the effects of matching. To calculate this peak the average response of each neuron across all trials and all conditions was calculated and then smoothed with a 20 ms boxcar. The peak was defined as the maximum value of the smoothed response. Responses during the time from 25 ms before to 25 ms after the peak were then analyzed. The mean response of the neuron in the match and catch conditions within this 50 ms box were calculated and compared using a randomized *t*-test.

## Results

The data reported in this paper were originally collected for another purpose and were published as part of other studies (Hayden and Gallant, 2005, 2009; David et al., 2008). However, the analyses presented here are all new. We recorded responses of 110 single-neurons in area V4 (100 in subject 1 and 10 in subject 2) while subjects performed a delayed match-to-sample task (Figure 1; Hayden and Gallant, 2005). On each trial, two rapidly changing streams of images appeared on opposite sides of a central fixation spot (Figure 1, see also Video S1 in Supplementary Material). The subject was rewarded for releasing a bar when a cued image (the target) appeared in the spatially cued stream. Two randomly chosen images were used as targets each day; new target images were chosen every day. To minimize any possible effects of arousal or satiation, we changed task conditions in 10-trial blocks; the spatial cue alternated each block; the cued image alternated each two blocks. Because we collected a minimum of 200 trials per neuron, we collected a minimum of 10 blocks for the feature comparison and 20 blocks for the spatial comparison.

To begin each trial, the subject touched and held a response bar and then fixated on a central spot. One spatial cue and one image cue then appeared simultaneously at fixation. The spatial cue indicated which one of the two image stream locations should be attended. The image cue thus indicated which of two possible images was the target (and thus the match stimulus) on a given trial. Following a brief delay, two rapidly changing streams (4 Hz) of up to 20 distractor images appeared, one at the cued location and one in the opposite hemifield. Reward was given if the response bar was released less than 1 s after the target image appeared in the spatially cued stream (hereafter called the match). The target was equally likely to occur at any of the 20 positions in the sequence of distractors. The target appeared once in the cued stream in all trials.

Inspection of the distribution of reaction times showed that subjects nearly always released the bar between 280 and 380 ms following the appearance of the match (>96% of trials). Median reaction time was 323 ms for subject 1 and 342 ms for subject 2. Subjects successfully maintained fixation on about 75% of trials, and on these trials they almost always responded to the correct target (subject 1: 98%, subject 2: 96%). Of the few errors that did not consist of broken saccades, most (89%) occurred when subjects released the bar following the appearance of the cued image at the spatially uncued location (hereafter called the spatial catch) while the rest (11%) occurred following the appearance of the uncued image at the spatially cued location (hereafter called the feature catch). Spatial and feature catches were presented with the same frequency as the match, so they occurred once per trial on average.

### Delay period response rates in V4 depend on cued images

**Figure 2:**
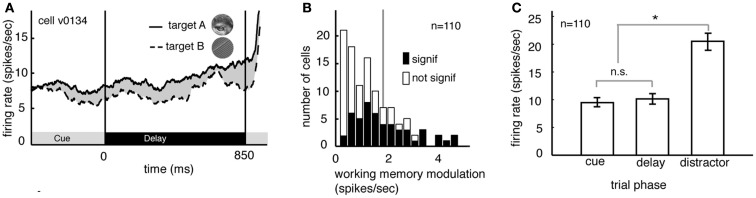
**Delay modulation in area V4 neurons depend on the remembered target image**. **(A)** Average responses of a typical neuron during the cue and delay periods for two memory conditions (the two conditions correspond to the two images shown in inset). Here responses are aligned to the beginning of the delay period and averaged over the two spatial conditions. The black bar below the curve denotes the time period used in the analysis. During the delay period the response rate was significantly greater when image A was the match (solid line) than when image B was the match (dashed line). **(B)** Image-dependent delay modulation histogram for our sample of 110 V4 neurons. Black bars denote significantly modulated cells. Vertical line indicates the mean modulation in significantly modulated cells. Significant delay modulation is more frequent than would be expected by chance. **(C)** Bar graph shows the average response of all neurons in our sample during the cue, delay, and early distractor portions of the task, averaged over all trial conditions. Responses during the cue and delay periods of the task are not significantly different. The lack of a visual response supports the idea that neurons are not activated by the cue. Cues do not evoke a sensory response in our task.

Several studies have suggested that delay period activity in V4 neurons reflects remembered or attended stimuli (Haenny et al., 1988; Maunsell et al., 1991; Luck et al., 1997). We compared delay period response rates obtained under the two different image target conditions, averaging over the two spatial conditions. Figure 2A illustrates the rates obtained from a single V4 neuron. (Responses are aligned to the beginning of the delay period.) On trials when image A was remembered, the neuron fired significantly more than on trials when image B was remembered (8.9 spikes/s versus 7.8 spikes/s, *p* < 0.02, randomized *t*-test).

Figure 2B summarizes the delay period response modulations for all 110 neurons in our sample. Response rates were significantly modulated by the identity of the remembered image in almost half of all cells (48.2%, *n* = 49/100 in subject V and 4/10 in subject G, *p* < 0.05, randomized *t*-test; data are collapsed over both spatial conditions). This number is significantly greater than would be expected by chance in the pooled data (which would be 5%, or 5.5 neurons, binomial test, *p* < 0.001). The size of the delay modulation for the subset of V4 neurons that showed significant effects is nearly 20% of the average delay response rate (modulation is 1.89 spikes/s on average). Although it is a small effect, it modulates a weak baseline (i.e., undriven) effect (about 10 spikes/s). Thus, as a proportion of the baseline, the magnitude of this effect is roughly equivalent to the ∼20% magnitude of attentional modulation of driven responses typically reported in area V4 (Mehta et al., 2000; Maunsell and Cook, 2002).

In many studies, one possible alternative explanation for the delay modulation that we observed is that it reflects long-lasting responses to the visual stimuli themselves. This explanation is not valid for the present study because image cues did not drive neural activity in the first place. Indeed, they appeared at the fixation point, far from the receptive fields of recorded neurons (cf. Romo et al., 2002; Bisley et al., 2004). Because the image cues did not produce observable phasic onset responses in our neurons (Figure 2C) it appears that this manipulation was successful. The average response rate of neurons to the sample was no different during the cue epoch and the delay epoch (*p* = 0.39, randomized *t*-test, Figure 2C). Moreover, significant differences between response rates during the cue and delay epoch were found in only a small minority of individual neurons (*n* = 6/100 in Subject V and 0/10 in subject G, *p* < 0.05, randomized *t*-test). This is the same number that would be expected by chance (*p* = 0.82, binomial test).

**Figure 3:**
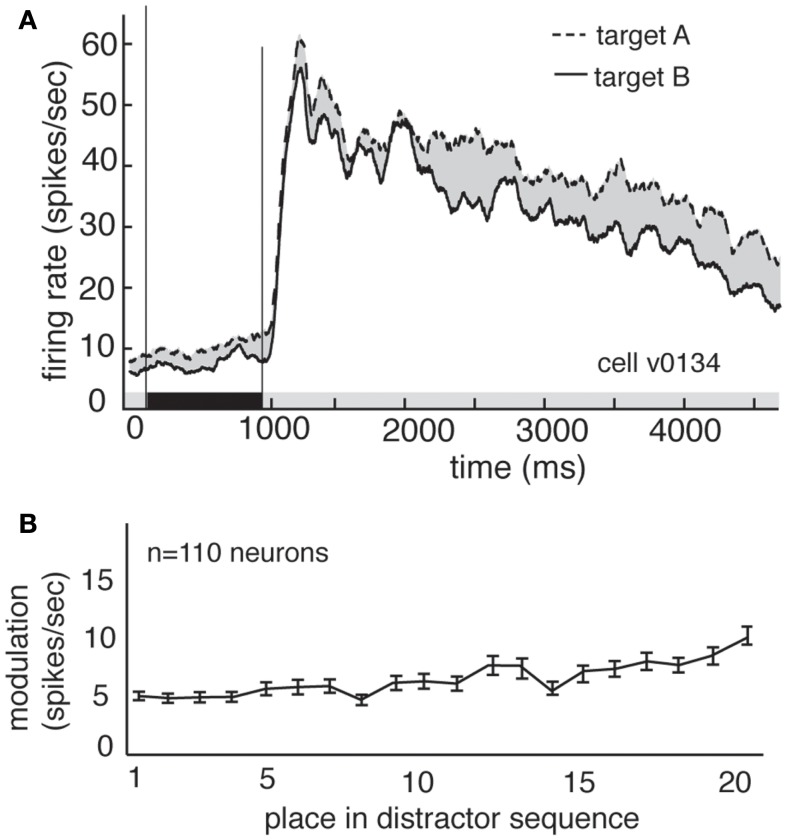
**Delay modulation persists across distractors**. **(A)** Average responses of the same neuron shown in Figure 2 during cue, delay, and distractor periods of the task. Delay modulation persists into distractor period and continues throughout distractor period for this neuron. Black bar below graph indicates delay period. **(B)** Size of modulation at each position in the distractor sequence, averaged over all trials and neurons. Bars indicate one standard error. Modulation is not abolished, even after 20 distractors.

Delay modulation may or may not reflect information storage in working memory. If delay modulation instantiates a memory trace that mediates detection of the match, then it should persist as long as the relevant information is remembered; persistent modulation is thus generally taken as a criterion for working memory signals (Constantinidis and Steinmetz, 1996; Miller et al., 1996; Moody et al., 1998). We therefore tested whether delay modulation persists after the delay and into the distractor period of the task (i.e., the rapid serial visual presentation, or RSVP). Responses of a single V4 neuron during the delay and distractor periods are illustrated in Figure 3A. During the delay period the response rate is low, but it increases rapidly once the first image appears in the cued stream. The response rate remains elevated the entire time that the stream is present, though it declines somewhat with each subsequent image (likely due to adaptation). In contrast, attentional modulation does not decline with each subsequent image. Figure 3B shows the average size of the modulatory effect of the remembered stimulus across all neurons. In both cases, delay period modulation persists during the distractor period, where it appears as a tonic enhancement of the visually evoked response (5.05 spikes/s at the beginning of the trial to more than 8.2 spikes/s by the end of the trial). This effect is significant (regression of response rate against image number, *p* < 0.001). This gradual enhancement may reflect a growing anticipation of reward (Shidara and Richmond, 2002; Sugrue et al., 2004, 2005).

Previous studies have shown that neural activity during the delay period of attention tasks is also modulated by remembered or attended spatial locations (Luck et al., 1997). We observe the same pattern. To quantify the effects of spatial attention/working memory, we compared firing in the two spatial conditions (attend in and attend out), averaging over the two feature attention conditions (image A and image B). The locus of spatial attention modulates delay activity in single V4 neurons (1.34 spikes/s on average, 2.45 spikes/s in significantly modulated neurons). This is about 15% of the average baseline response rate (9.1 spikes/s). The size of this effect is substantially larger in the subset of neurons that show a significant modulation of visual responses for spatial attention, amounting to 27% of the baseline response rate. Furthermore, the modulation produced by spatial and image cues is similar (1.34 spikes/s for spatial cues vs 1.89 spikes/s for image cues; these effects are not significantly different, *p* = 0.16, randomized *t*-test).

**Figure 4:**
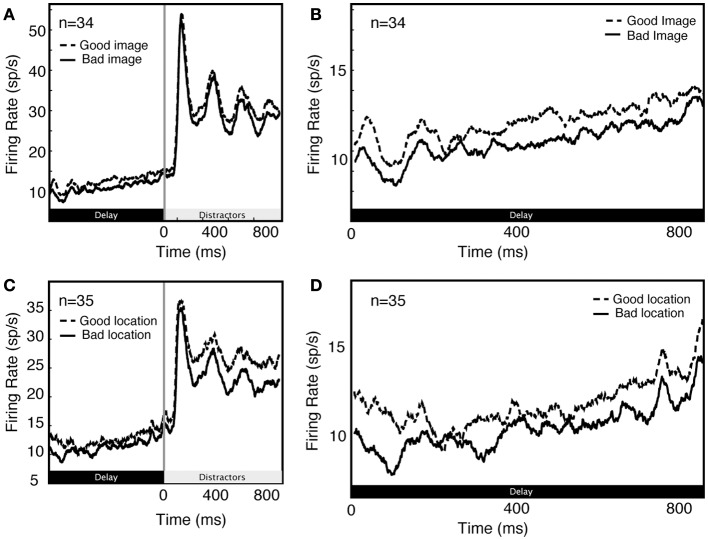
**Comparison of feature **(A,B)** and spatial **(C,D)** effects during early part of trial **(A,C)** and during delay period along **(B,D)****.

Unsurprisingly, we found that delay activity depends on spatial attention as well as feature-based attention (Figure 4). As these effects are well described in the literature, they were not a focus of the present set of analyses.

### Responses to the match image are greater than responses to feature catches

Match enhancement is a potential basis for memory-guided decision-making because these enhanced response provide enough information to guide correct bar release (Haenny et al., 1988; Maunsell et al., 1991; Riches et al., 1991; Eskandar et al., 1992; Miller and Desimone, 1994; Vogels et al., 1995; Miller et al., 1996; Romo and Salinas, 2001; Ogawa and Komatsu, 2004, 2006; Bichot et al., 2005; Mirabella et al., 2007). To determine whether V4 neurons show match enhancement, we compared neural responses evoked by match images to those evoked by feature catch images. Because we used the same two images as matches and as feature catches (in different blocks of trials), this analysis allows us to compare responses to the same two images in different behavioral contexts. (Note that on these trials spatial attention was always directed toward the receptive field of the recorded neuron; other trials were excluded from this analysis).

**Figure 5:**
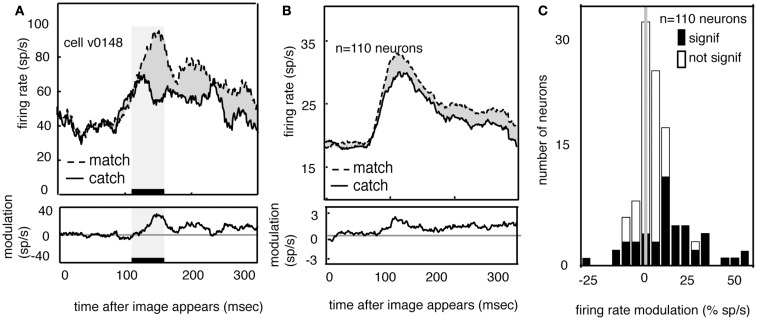
**Neural responses to images are enhanced when the appear in the match context**. **(A)** Average response of a typical neuron to matches and feature catches, and their difference. Responses are enhanced when the stimulus matches the one held in memory. This enhancement appears during the early portion of the response (black bar beneath curve). **(B)** Average response of all neurons in our sample (*n* = 110) in match and catch conditions, and their difference. Across the population, responses to the match are greater than responses to the feature catch. The size of this modulation is relatively consistent across the response. **(C)** Histogram of the size of match enhancement for all the neurons in our sample. Black bars denote significantly modulated neurons. Population is significantly skewed to the right (*p* < 0.01), indicating that match enhancement is significantly more common than suppression.

Figure 5A shows the average response of one V4 neuron to the two match images (dashed line) and to the two feature catch images (solid). Responses are aligned to the time of the appearance of the image in the receptive field. Neuronal responses to the match image are greater than responses to the feature catch image. The difference (10 spikes/s) is 15% of the response rate (*p* < 0.01, randomized *t*-test). The average responses of all 110 V4 neurons in our sample to the match and feature catch images are shown in Figure 5B. A histogram of individual effects, normalized to the catch response, is shown in Figure 5C. Responses to the match and feature catch image differ significantly in nearly half these neurons (42/100 in subject V and 6/10 in subject G, *p* < 0.05, 44%, randomized *t*-test). Among neurons showing significant match-related modulation, match enhancement is more frequent than match suppression (37/48 show match enhancement). This difference is significant (*p* < 0.001, binomial test). One quarter of the neurons in our sample show both persistent delay modulation and match enhancement (*n* = 25/100 in subject V and 3/10 in subject G). This number is larger than would be expected by chance (binomial test, *p* > 0.05). The existence of some neurons that show both delay modulation and match enhancement is consistent with the idea that delay modulation and match enhancement reflect a common underlying process. Note, however, that this overlap in effects was not observed in all modulated neurons.

If match enhancement reflects a comparison process that occurs within V4, then it should affect the early portion of the visual response, before feedback signals have time to arrive from more central cortical areas. On the other hand, if match enhancement reflects a comparison process that occurs in the prefrontal cortex, it should only appear during the later portion of the visual response (Ferrera et al., 1994; Miller and Desimone, 1994; Chelazzi et al., 1998; Lamme and Roelfsema, 2000; Ogawa and Komatsu, 2006; Mirabella et al., 2007). To investigate this issue we compared responses to match and catch images in a 50 ms epoch beginning 100 ms after stimulus onset (this period is indicated by the black bar and shaded rectangle in Figure 5A). The average latency of the peak response in our sample of V4 neurons was about 120 ms, so this epoch is approximately centered on the peak of the average early response transient. We focused on this early epoch because the transient response is generally assumed to represent the first volley of feed-forward information (VanRullen and Thorpe, 2002). Modulation in this early time window is significant in over half the neurons that show a significant match effect (54%, *n* = 26/48, *p* < 0.05, randomized *t*-test).

### Responses to spatial catch images are greater than responses to other images

Feature-based attention affects responses of neurons across the visual field, not just at the attended location (Martinez-Trujillo and Treue, 2004; David et al., 2008; Hayden and Gallant, 2009). This fact suggests that when a particular visual stimulus is relevant, neurons across the visual field change their response properties. We therefore hypothesized that we would observe changes in response properties of visual neurons with receptive fields that lay outside the locus of spatial attention. To investigate this issue we examined how V4 neurons respond to remembered stimuli when they are presented away from the cued location – and thus need to be ignored. Here we call these spatial catch images. Because the two target images used each day were chosen at random without regard to the tuning of recorded neurons, any difference in neuronal responses between the spatial catches and other distractors would be unlikely to arise by chance.

**Figure 6:**
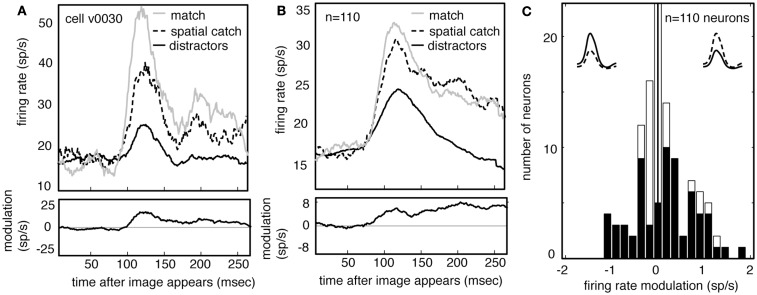
**Neuronal responses are enhanced when images appear in the spatial catch context**. **(A)** Average response of a typical neuron to spatial catches (dashed) and distractors (solid), and their difference. Responses are enhanced when the stimulus matches the one held in memory, even when it appears at the unattended location and does not lead to a motor response. For comparison, responses to matches are shown as well (gray). **(B)** Average response of all neurons in our sample (*n* = 110) in catch and distractor conditions, and their difference. Across the population, responses to the catch are greater than responses to the distractors. The size of this modulation is relatively consistent across the response. Response to match is shown for comparison (gray). **(C)** Histogram of the size of catch enhancement for all the neurons in our sample. Black bars denote significantly modulated neurons. Population is significantly skewed to the right (*p* < 0.01), indicating that match enhancement is significantly more common than suppression.

Figure 6A shows the average responses of one V4 neuron (same neuron as in Figure 5) to all spatial catches (dashed line) and to other distractors (solid black line). Responses to the spatial catch are significantly greater than responses to distractors (difference is 6.4 spikes/s, *p* < 0.01, randomized *t*-test). The same pattern is observed in the population activity (Figure 6B, difference is 5.1 spikes/s, *p* < 0.01, randomized *t*-test). Responses to the spatial catch are significantly different from responses to the distractors in the majority of neurons (*n* = 61/100 in subject V, 6/10 in subject G, *p* < 0.05, randomized *t*-test). In these neurons, there is a non-significant trend toward enhancement being more prevalent than suppression (Figure 6C; *n* = 41/67 neurons, *p* = 0.086, binomial test). The modulated responses to spatial catches suggests that the average neuronal response to the remembered images are affected at locations away from the locus of attention during memory-guided decision-making.

Because our subjects typically (and correctly) ignored these spatial catches, the response modulation for spatial catches rules out alternative explanations for match enhancement for match images, including reward expectation or motor planning. Instead, these data indicate that maintaining an image in working memory for the purposes of this task alters the visual response properties of the neurons themselves.

## Discussion

Comparing a stimulus to one held in working memory is a hallmark of higher cognition (Desimone, 1996; Romo and Salinas, 2003; Machens et al., 2005; Pasternak and Greenlee, 2005; Mirabella et al., 2007; Lui and Pasternak, 2011). Standard models of memory-guided decision-making hold that a representation of the remembered stimulus is maintained within the lateral prefrontal cortex in the form of changes to the tonic response rates of neurons (Funahashi et al., 1989; Goldman-Rakic, 1995; Desimone, 1996; Miller et al., 1996; Rainer et al., 1998). These same explanations hold that decision-making reflects the outcome of comparison processes that occur within the frontal cortex (Hernandez et al., 2010; Romo et al., 2002; Machens et al., 2005; Miller and Wang, 2006; Jun et al., 2010). These models are consistent with the established primacy of the prefrontal cortex in executive control, and thus assume that working memory and decision processes are anatomically and computationally distinct from perception, which is thought to be localized to caudal sensory and association areas.

Here we considered the alternative possibility that visual cortex participates in both the storage and comparison aspects of memory-guided decision-making, beyond its well-established perceptual aspects. We were particularly interested in testing the idea that V4 neurons act as matched filters. The matched filter hypothesis holds that selective attention shifts the tuning properties of sensory and association neurons so that they more closely approximate the searched-for stimulus (Olshausen et al., 1993; Motter, 1994; Lee et al., 1999; Rao and Ballard, 1999; Cutzu and Tsotsos, 2003; Carrasco et al., 2004; Lu and Dosher, 2004; Machens et al., 2005; Compte and Wang, 2006; Mirabella et al., 2007; David et al., 2008; Sugase-Miyamoto et al., 2008). This hypothesis holds that V4 neurons change their tuning when the cues are presented, and this change in tuning in turn enhances the response of the neuron to the match when it appears in the sequence of distractors, and that the output of V4 is therefore sufficient to guide the appropriate action without any additional need for storage or comparison processes.

According to this hypothesis neural responses should increase when a stimulus on the monitor is similar to the remembered one, and should decrease when the stimulus on the monitor is dissimilar to the remembered one (Sugase-Miyamoto et al., 2008). A matched filter mechanism for memory-guided decision-making is efficient: since the same neurons are used for representation, storage, and decision-making, there is no need to for the brain to reproduce a representational schema in the prefrontal cortex. A second benefit to a matched filter mechanism is that it reduces the computational demands of downstream neurons. The outputs of V4, after filtering for noise, have sufficient information to drive behavior directly without any additional transformations.

We find that response patterns of V4 neurons exhibit three properties consistent with the matched filter hypothesis. First, between the cue and the appearance of the match, response rates depend on the contents of working memory, suggesting that these neurons’ basal response properties are altered by the identity of the remembered cue, and that they participate in storage. Second, when the remembered cue reappears in the stream of distractors, responses are enhanced, suggesting that these neurons maintain a memory of the match. Third, responses to remembered images that appear at the unattended location are greater than responses to other distractors, suggesting that tuning shifts apply to neurons across the visual field. These results are consistent with our previous results indicating that tuning properties of V4 neurons can change based on task demands (David et al., 2008). It is worth pointing out that, regardless of condition, firing rate declines with distractor number (Figure 3A). The size of this effect is greater than the size of both the memory-related modulation and match enhancement; consequently, any decoder must adjust for this effect, perhaps by normalizing to the average firing rate of the cell, or by receiving a parallel signal indicating progress through the trial.

If memory-guided visual search does change the tuning properties of V4 neurons then feature-based attention and task-relevant match enhancements may turn out to be two sides of the same coin. According to the matched filter model the tuning function of each neuron shifts toward the remembered stimulus. Simpler forms of modulation, such a changes in the baseline response or response gain, do not constitute a matched filter mechanism. Thus, the matched filter hypothesis is a more complicated and powerful form of attentional modulation than has been shown in previous studies that reported attentional modulation of response baseline (Luck et al., 1997), response gain (McAdams and Maunsell, 1999), sensitivity (Reynolds et al., 2000), or selectivity (Spitzer et al., 1988).

In summary, our data are consistent with the idea that individual neurons in area V4 serve as matched filters. However, these data are not sufficient to confirm that area V4 instantiates a matched filter. Furthermore, we have no evidence that area V4 is the only visual area that embodies a matched filter, and in fact we suspect that V4 is one of many areas that do so. Finally, our data do not exclude a role for the prefrontal cortex in storage and comparison. In fact, we suspect that memory-guided decision-making involves multiple brain regions acting in concert.

The present results are similar in some respects to those reported in an earlier study of the relationship between top-down and bottom-up attentional effects in V4 (Ogawa and Komatsu, 2004). Subjects in that study were rewarded for selecting one of two oddball stimuli that varied on a specified dimension. Responses of V4 neurons were enhanced to the behaviorally relevant stimuli, consistent with the present results. A follow-up study showed that neuronal response rates in area V4 initially signified low-level (bottom-up) stimulus properties, but later signaled behavioral relevance (Ogawa and Komatsu, 2006). These authors remained agnostic about whether the observed changes were prepotent (i.e., the control was proactive), but the presence of small modulations early in the visual response is consistent with this idea.

Chelazzi et al. (1998) showed that late portions of visual responses in IT reflect attended stimuli. Building on this work, a recent study examined the effects of behavioral relevance of visual stimuli on responses of V4 neurons (Mirabella et al., 2007), and found that about one third of neurons showed a similar pattern of attentional modulation. Mirabella et al. also reported a delay effect of feature-based attention. The present results confirm these earlier results, and extend them in three important ways. First, we show that these modulatory effects can emerge as early as 50 ms after the appearance of an image, thus providing stronger evidence that prepotent modulations of V4 response properties mediate the observed enhancements. Second, by controlling both the remembered cue and the attended location, we were able to show that modulatory effects occur outside the locus of spatial attention. Finally, our rapid serial visual search task design allowed us to show that modulatory activity persists across irrelevant distractors, providing stronger evidence that the observed modulations may embody a working memory signal. In sum, the two studies broadly agree and Mirabella et al. argued, as we do, that adjustments to tuning properties of V4 neurons may simultaneously embody perception, working memory, and decision-making processes. The present results therefore provide further evidence that visual cortex dynamically adjusts its tuning to enhance performance (Allport et al., 1994; Rao and Ballard, 1999). Moreover, these results point to a potential basis for memory-guided decision-making that eschews traditional notions of discrete cognitive processes of perception, working memory, comparison, and decision, in favor of a single parsimonious mechanism (Mirabella et al., 2007).

## Conflict of Interest Statement

The authors declare that the research was conducted in the absence of any commercial or financial relationships that could be construed as a potential conflict of interest.

## Supplementary Material

The Supplementary Material for this article can be found online at http://www.frontiersin.org/Decision_Neuroscience/10.3389/fnins.2013.00018/abstract
